# Supplementary material for: Gravity measurements below 10−9g with a transportable absolute quantum gravimeter
Source: Sci Rep. 2018 Aug 17;8:12300. doi: 10.1038/s41598-018-30608-1 (PMC6098009; doi:10.1038/s41598-018-30608-1)
Supplement: Supplementary file 1 — Supplementary material [file 41598_2018_30608_MOESM1_ESM.pdf]

Gravity measurements below 10^{-9} g with a transportable absolute quantum gravimeter: Supplementary material

Vincent Ménoret[1], Pierre Vermeulen[1], Nicolas Le Moigne[2], Sylvain Bonvalot[3], Philippe Bouyer[4], Arnaud Landragin[5], and Bruno Desruelle[1,*]

[1]MUQUANS, Institut d'Optique d'Aquitaine, rue François Mitterrand, 33400, Talence, France
[2]Géosciences Montpellier, CNRS, Université Montpellier, UA, F-34095 Montpellier, France
[3]GET, IRD, CNRS, CNES, Université de Toulouse, F–31400 Toulouse, France
[4]LP2N, Laboratoire de Photonique Numérique et Nanosciences, Institut d'Optique Graduate School, rue François Mitterrand, 33400 Talence, France
[5]LNE–SYRTE, Observatoire de Paris, Université PSL, CNRS, Sorbonne Université, 61 avenue de l'Observatoire, F–75014 Paris, France
[*]bruno.desruelle@muquans.com

Supplementary Methods: AQG measurement principle and sensitivity estimation

We describe the measurement principle of the Absolute Quantum Gravimeter in a simplified way, so as to try and give an intuitive physical explanation. A full description can be found in the references. We illustrate the measurement principle with calculations of the signal expected for the parameters given in the text, chosen to be representative of the Absolute Quantum Gravimeter.

Measurement sequence and origin of the phase shift

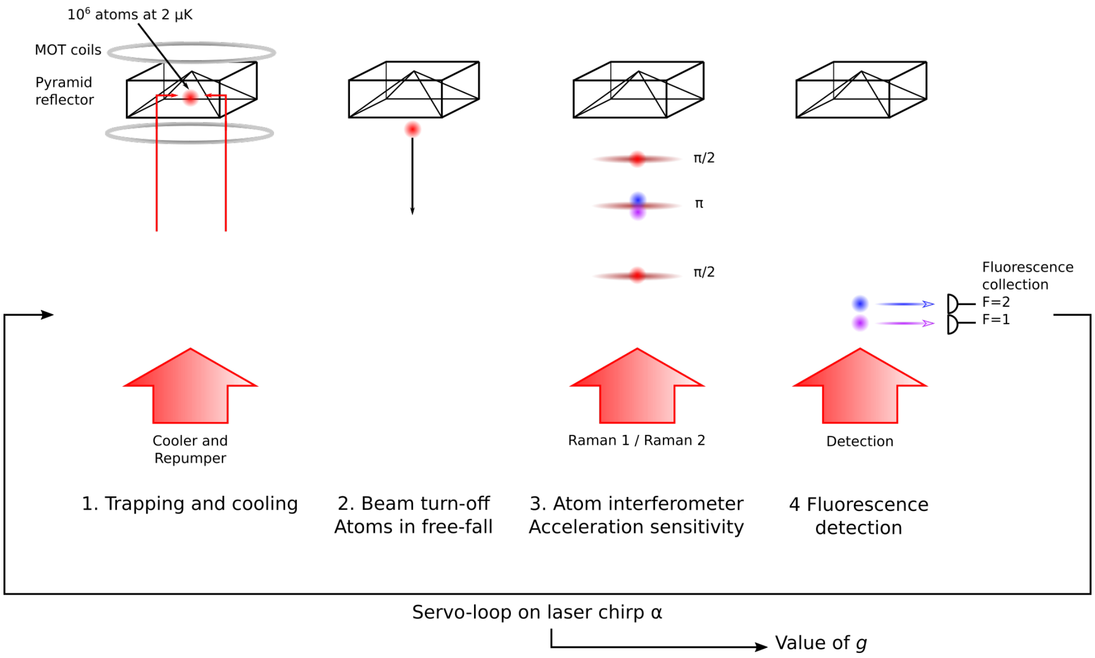

Figure S1. AQG measurement sequence. **1.** Atoms are magneto-optically trapped, and cooled to 2 μK in an optical molasses. After preparation, there are approximately 10^6 atoms in the $|F=1\rangle$ state. **2.** The lasers are turned off and the atoms are in free-fall. **3.** During the free-fall, a sequence of three laser pulses is applied to measure the acceleration of the atoms as they fall. **4.** At the bottom of the chamber, we use fluorescence detection to count the number of atoms in each internal state.

The AQG measures gravity with a cycling frequency close to 2 Hz. At each cycle (see Fig. S1), approximately 10^7 ^{87}Rb atoms are trapped in the pyramid reflector at the top of the vacuum chamber. They are further cooled to below 2 μK in an optical

molasses. At this stage, the atoms are equally distributed between the 5 Zeeman sublevels of $|5^2S_{1/2}, F = 1\rangle$. We select the atoms in the magnetically insensitive $m_F = 0$ state and end up with approximately 10^6 atoms in the $|5^2S_{1/2}, F = 1, m_F = 0\rangle$ level[1] (see [1] for a diagram of the energy levels of ^{87}Rb and all the relevant information about this atom and the spectroscopy of its D_2 line). These atoms are in a cloud of a few hundred micrometers in diameter, at a temperature close to absolute zero. At this temperature, each atom cannot be described as a single particle anymore, but as a short burst of localized matter wave (a wavepacket) whose center will evolve as its classical alter-ego, following a free-fall trajectory under the influence of gravity. An atom interferometry sequence of three pulsed stimulated Raman transitions of durations 10, 20 and 10 μs in a $\pi/2 - \pi - \pi/2$ configuration is used to perform the gravimetric measurement [2]. This type of sequence has been extensively studied and used for precision measurements [3, 4, 5]. The first laser pulse ($\pi/2$) splits the wave packet in two copies with a slight slowing down of the fall of one of the copies, thus creating two different paths corresponding to the two arms of an interferometer. The second laser pulse (π) slows down the fall of the second copy and accelerates the fall of the first one, so that they meet again and close the interferometer. The last laser pulse ($\pi/2$) merges the copies when they meet to observe the interference that would result from a phase difference between them (Fig. S2). Each output port of the interferometer is labeled by a single atomic internal state $|F = 1\rangle$ or $|F = 2\rangle$ [6]. By using state selective fluorescence detection at the bottom of the chamber, we count the number of atoms in each level, record the interference pattern and measure the corresponding interferometric phase shift. The proportion of atoms in the $|F = 2\rangle$ state (also referred to as transition probability) is given by

$$P = 0.5 \times (1 - C \cos \Phi), \tag{1}$$

where C is the contrast of the fringes and Φ the interferometric phase shift.

Where does this phase shift come from? Whenever the light pulse – a standing wave produced by the interference of two laser waves propagating in opposite directions – alters the fall of a wavepacket copy, it induces on this copy a slight phase shift proportional to $4\pi/\lambda \times x$, where λ is the wavelength of the laser and x the position of the center of the wave packet (i.e. the classical center of mass position of the atom [7]) in the laser wave, and whose sign will change depending on whether the fall of the wavepacket copy is slowed down or accelerated. The final phase shift of the interferometer is then directly proportional to the distance travelled by the wave packet between the first and the last pulse:

$$\Phi = \frac{4\pi}{\lambda} g T^2, \tag{2}$$

where T is the interrogation time between the light pulses.

Compensation of Doppler effect

In practice, the stimulated Raman transitions use two lasers tuned close to the optical transition at $\lambda = 780$ nm, that propagate in opposite directions. If the frequency difference $\Delta f_{\mathrm{Raman}}$ between the two lasers is equal to the Rb clock transition, i.e. the frequency difference between the internal states $|F = 1\rangle$ and $|F = 2\rangle$, these two lasers coherently drive a transition between the two states that is accompanied by a net slowing down or acceleration of the fall by a quantity $\hbar k_{\mathrm{eff}}/M$, where $k_{\mathrm{eff}} = 2 \times 2\pi/\lambda \approx 16 \times 10^6$ m^{-1} is the effective two-photon Raman wavevector and M the mass of the atoms. This effect is what causes the wavepacket copies described above to accelerate or slow down, and builds the interferometer.

As the lasers propagate in opposite directions, their frequency is shifted by Doppler effect if the atoms have a non-zero velocity. Consequently, the Raman pulses are highly velocity selective [8], which leads to a strong shift of the resonance condition by a quantity

$$\Delta f_{\mathrm{D}} = \frac{\vec{k_{\mathrm{eff}}}.\vec{v}}{2\pi} = \frac{k_{\mathrm{eff}} g t}{2\pi}, \tag{3}$$

with v the mean atomic velocity and t the time since the beginning of the free-fall. The velocity selection effect also results in a reduced contrast C of the interferometer.

Because the three pulses are separated in time, this Doppler shift Δf_{D} is different for each of them. This is compensated by applying an adjustable chirp of parameter α to the frequency difference $\Delta f_{\mathrm{Raman}}$ between the two lasers:

$$\Delta f_{\mathrm{Raman}} = \Delta f_{\mathrm{Raman},t=0} + \alpha t. \tag{4}$$

If α is chosen so that the resonance condition is met for each pulse (i.e. perfect compensation of the Doppler effect), the dephasing in the interferometer vanishes [9, 10]. The specific value α_0 producing this null dephasing thus leads to a determination of the value of the acceleration g:

$$g = 2\pi \frac{\alpha_0}{k_{\mathrm{eff}}}. \tag{5}$$

[1] In the following, we simply label $|F = 1\rangle$ (resp. $|F = 2\rangle$) the $|5^2S_{1/2}, F = 1, m_F = 0\rangle$ (resp. $|5^2S_{1/2}, F = 2, m_F = 0\rangle$) state of the atom.

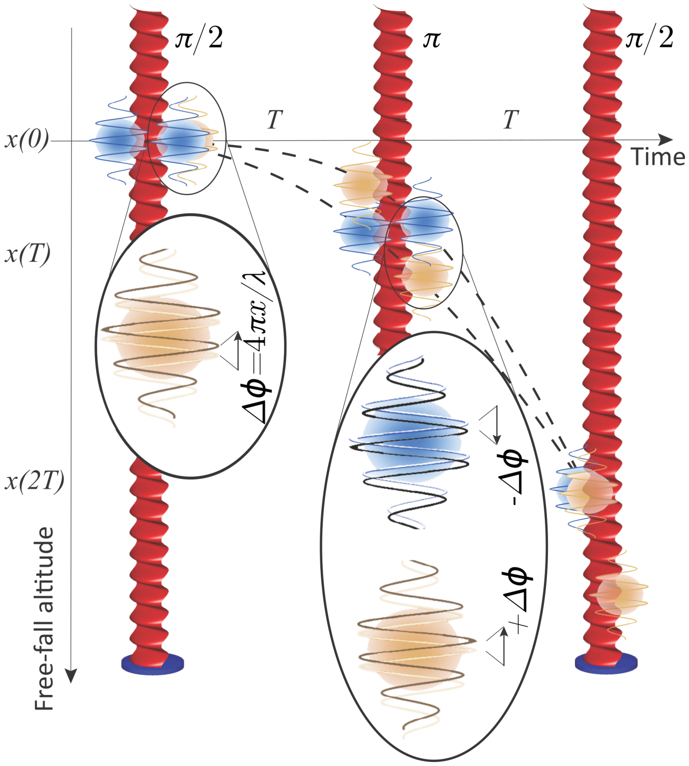

Figure S2. Principle of operation of a quantum gravimeter. A wavepacket initially prepared in the $|F=1\rangle$ state (blue), resulting from the cooling of atoms to a temperature close to absolute zero, is illuminated by 3 successive light pulses. Whenever the wave packet is modified by the pulse, its phase is changed by a quantity $\Delta\phi$ proportional to the position of its center of mass in the light wave. At $t=0$, the upshifted wave packet (brown, corresponding to $|F=2\rangle$) receives a phase shift of $\Delta\phi=+4\pi/\lambda\times x(0)$. At time T, this upper copy is shifted down and receives a phase shift of $-\Delta\phi=-4\pi/\lambda\times x(T)$ while the lower copy (blue, $|F=1\rangle$) is shifted up and receives a phase shift of $\Delta\phi=+4\pi/\lambda\times x(T)$. The last pulse, where the 2 copies merge again, transfers a phase shift $\Delta\phi=+4\pi/\lambda\times x(2T)$ to the upper copy. The resulting interferometer phase shift is the difference between the phase received by the upper copy and the lower copy, i.e. $(4\pi/\lambda)\times(x(0)-2x(T)+x(2T))$. As the atoms are in free-fall, this amounts to $4\pi/\lambda\times gT^2$.

Close to this value, the interferometer phase shift becomes

$$\Phi=\left(\frac{4\pi}{\lambda}g-2\pi\alpha\right)T^2=(k_{\text{eff}}g-2\pi\alpha)T^2. \tag{6}$$

By varying α from one shot to the next for a given interrogation time T, we can scan the interference pattern. To identify the null phaseshift where $k_{\text{eff}}g-2\pi\alpha_0=0$, we scan several sets of fringes for different values of T. The point where P is minimum for all the values of T corresponds to $\Phi=0$ (Fig. S3).

Practical implementation

In the AQG we use Rubidium atoms ($\lambda\approx 780$ nm), and T is of the order of 60 ms. The phase shift (Eq. (2)) is more than 5.6×10^5 radians for an acceleration of 9.81 m.s^{-2}. If we measure this phase shift with a resolution of 17 mrad (i.e. a detection signal-to-noise ratio of 150 and a contrast of 40% at the output of the interferometer), the minimum acceleration variation that can be observed will be of the order of 300 nm.s^{-2} (or approximately $3\times10^{-8}g$). Successive measurements, performed at a rate of 2 Hz, allow for an efficient averaging of the data. A relative sensitivity of 1×10^{-9} corresponds to an improvement by a factor 30 of the single-shot sensitivity. Assuming white noise, it is reached after averaging over $30^2=900$ shots, or 450 s. In the case of the AQG, sensitivity is not ultimately limited by the detection SNR, but by residual vibrations. The relative single shot sensitivity is of the order of 7×10^{-8}, so it takes approximately 2500 s to reach $1\times10^{-9}g$.

We start with a small interrogation time T_1. This way, the period of the fringes ($1/T_1^2$) is large enough to make sure we can determine g with low precision for any place on Earth. We choose $T_1=2$ ms and scan α for one period around 25 MHz.s^{-1} (corresponding to $g\approx9.81$ m.s^{-2}). This means we measure g with a relative sensitivity of 2.6×10^{-5} between 9.77 and 9.86 m.s^{-2}.

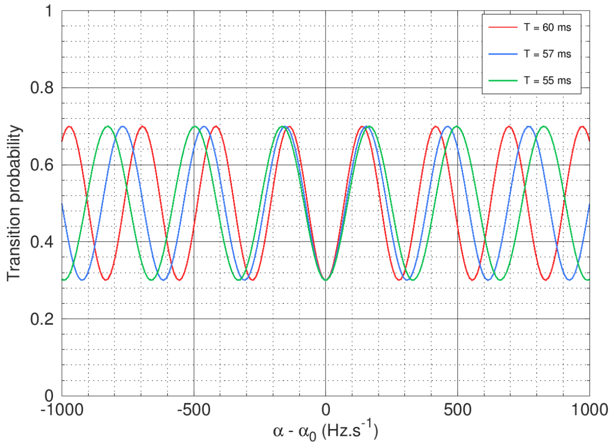

Figure S3. Interference fringes obtained with three values of T. The value of α where the transition probability P is minimum for all values of T corresponds to the null phase shift, where the frequency chirp exactly compensates the gravity induced Doppler effect.

We find the value α_{0,T_1} corresponding to the null phase shift. We increase T to $T_2 = 7$ ms and scan a fringe around α_{0,T_1}. Again, we find the null phase shift α_{0,T_2}, with an increased relative precision of 2.2×10^{-6} while making sure we are determining the value of g without ambiguity. We then increase T to $T_3 = 17$ ms to measure α_{0,T_3}, and finally to 60 ms and determine the final value α_0. The value of g is now known with a relative sensitivity of 3×10^{-8} as discussed above (Fig. S4).

Once this initial measurement has been performed, we start the servo-loop that maintains the frequency chirp α around the null phase shift. We briefly recall the process, which has been described in [11]. We alternate half-fringe measurements around α_0, and from the resulting values of transition probability P_i and P_{i+1} we can estimate a phase error. A correction $G \times (P_{i+1} - P_i)$ is applied to α at the next cycle to stir the chirp rate on the central fringe. This realizes an integrator, whose time constant can be set to a few cycles by adjusting the gain G. This locking technique has the advantage of rejecting offset and contrast fluctuations, while preserving maximal sensitivity to phase fluctuations.

We emphasize that the AQG operates in a sequential way and that several experimental cycles are necessary to compute a value of g. In this respect it is significantly different from optical absolute gravimeters, where a single drop is enough to determine g.

References

[1] D. A. Steck. Rubidium 87 D Line Data, 2015. http://steck.us/alkalidata Accessed: 2018-03-30.

[2] Mark Kasevich and Steven Chu. Atomic interferometry using stimulated Raman transitions. *Phys. Rev. Lett.*, 67:181–184, Jul 1991.

[3] A Peters, K Y Chung, and S Chu. Measurement of gravitational acceleration by dropping atoms. *Nature*, 400:849, 1999.

[4] P Gillot, O Francis, A Landragin, F Pereira Dos Santos, and S Merlet. Stability comparison of two absolute gravimeters: optical versus atomic interferometers. *Metrologia*, 51(5):L15, 2014.

[5] C Freier, M Hauth, V Schkolnik, B Leykauf, M Schilling, H Wziontek, H-G Scherneck, J Müller, and A Peters. Mobile quantum gravity sensor with unprecedented stability. *Journal of Physics: Conference Series*, 723(1):012050, 2016.

[6] Ch.J. Bordé. Atomic interferometry with internal state labelling. *Physics Letters A*, 140(1):10 – 12, 1989.

[7] Peter Asenbaum, Chris Overstreet, Tim Kovachy, Daniel D. Brown, Jason M. Hogan, and Mark A. Kasevich. Phase shift in an atom interferometer due to spacetime curvature across its wave function. *Phys. Rev. Lett.*, 118:183602, May 2017.

[8] Mark Kasevich, David S. Weiss, Erling Riis, Kathryn Moler, Steven Kasapi, and Steven Chu. Atomic velocity selection using stimulated Raman transitions. *Phys. Rev. Lett.*, 66:2297–2300, 06 1991.

[9] S Merlet, J Le Gouët, Q Bodart, A Clairon, A Landragin, F Pereira Dos Santos, and P Rouchon. Operating an atom interferometer beyond its linear range. *Metrologia*, 46(1):87, 2009.

[10] M-T Jaekel, B Lamine, and S Reynaud. Phases and relativity in atomic gravimetry. *Classical and Quantum Gravity*, 30(6):065006, 2013.

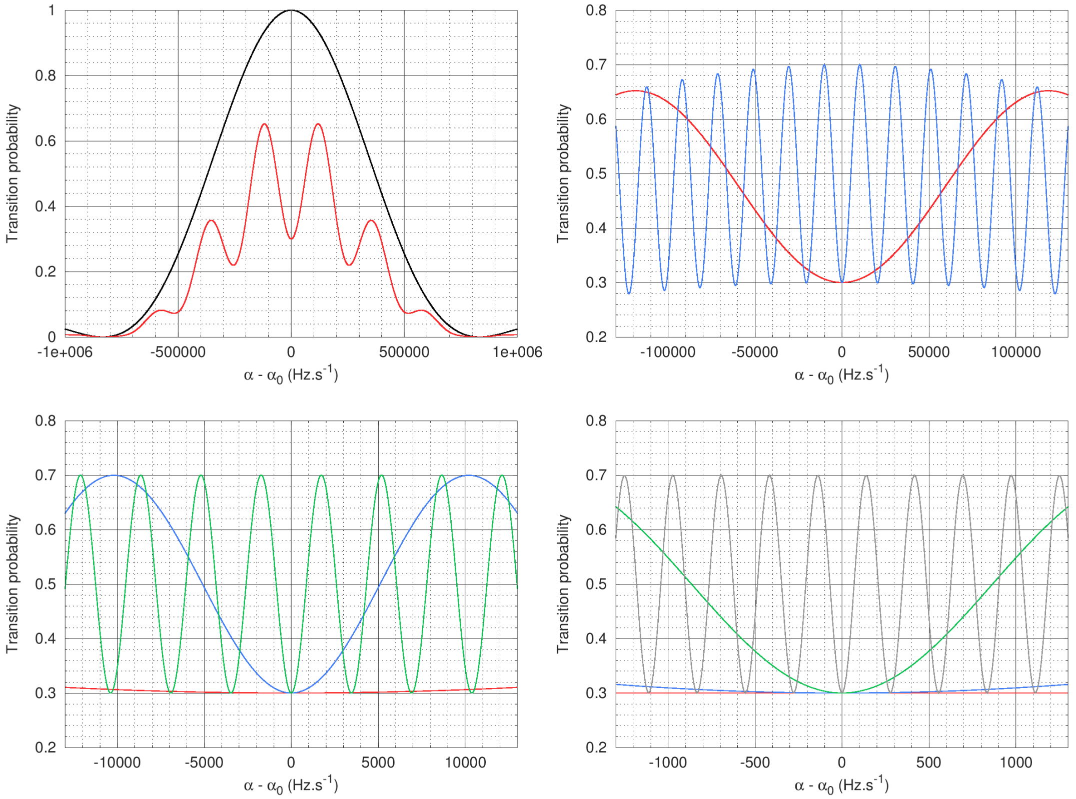

Figure S4. Sequence performed to initiate the measurement of g. Fringes are scanned with increasing values of T in order to measure g with no ambiguity. Red: $T_1 = 2$ ms. Blue: $T_2 = 7$ ms. Green: $T_3 = 17$ ms. Gray: $T = 60$ ms. The black curve in the top left plot shows the envelope of the spectrum, determined by the Fourier transform of a single Raman pulse of duration τ.

[11] Anne Louchet-Chauvet, Tristan Farah, Quentin Bodart, André Clairon, Arnaud Landragin, Sébastien Merlet, and Franck Pereira Dos Santos. The influence of transverse motion within an atomic gravimeter. *New Journal of Physics*, 13(6):065025, 2011.
